# The association of serum adiponectin with abdominal aortic calcification in Japanese male hemodialysis patients: a cross-sectional observational study

**DOI:** 10.1038/s41598-017-06850-4

**Published:** 2017-07-25

**Authors:** Takeshi Sakura, Senji Okuno, Eriko Nishio, Kyoko Norimine, Eiji Ishimura, Tomoyuki Yamakawa, Shigeichi Shoji, Masaaki Inaba

**Affiliations:** 1 Department of Metabolism, Endocrinology and Molecular Medicine, and Department of Nephrology, Osaka City University Graduate School of Medicine, Osaka, Japan; 2 Kidney Center, Shirasagi Hospital, Osaka, Japan

## Abstract

The negative relation of serum adiponectin to atherosclerosis becomes a positive association in patients with chronic kidney disease (CKD). We conducted a small-scale cross-sectional observational study, in 101 Japanese male hemodialysis patients, to examine the relationship of serum adiponectin and leptin to abdominal aortic calcification (AAC). The presence of AAC was evaluated from simple X-ray radiographs of the left lateral abdomen. Serum adiponectin was significantly higher in AAC-positive patients [18.8 (13.0–28.1) μg/mL] than in AAC-negative patients [15.4 (8.9–22.8) μg/mL] (p = 0.03), whereas serum leptin did not differ significantly between the two groups. Multiple logistic regression analysis showed that log adiponectin, but not log leptin, was independently and significantly associated in a positive manner with AAC (odds ratio: 16.31, 95% confidence interval: 1.70–156.41, p = 0.02), after adjustment for age, body weight, percentage body fat, hemodialysis duration, prevalence of diabetes mellitus, and other risk factors. In conclusion, we found a positive and independent association of serum adiponectin with AAC in male hemodialysis patients, indicating that the reversed association between serum adiponectin and atherosclerosis in patients with CKD dose not result from increased serum adiponectin due to the impaired urinary secretion.

## Introduction

Adiponectin, a 244-amino acid protein, is an adipokine synthesized by adipocytes. Serum adiponectin level decreases in the subjects with a high risk for cardiovascular disease (CVD), such as those with obesity^1^, diabetes mellitus (DM), coronary artery disease, and dyslipidemia. Recent findings have demonstrated a significant inverse association of serum adiponectin with CVD risk in both the general population and patients with DM, establishing the hypothesis that a higher serum adiponectin level might be predictive of a lower CVD risk^2^. However, the negative correlation between serum adiponectin and CVD risk is converted to a positive correlation in pre-dialysis chronic kidney disease (CKD) patients, because (i) pre-dialysis CKD patients show higher serum adiponectin due to impaired urinary adiponectin excretion^3^, and (ii) CKD is a definite CVD risk factor in the Japanese population^4^. The positive association of increased serum adiponectin with the presence of abdominal aortic calcification (AAC) has been reported in pre-dialysis CKD patients. Therefore, by investigating the association between the two parameters in hemodialysis patients who do not have residual renal function, it may be possible to determine whether the conversion from a negative to a positive association between serum adiponectin and AAC in pre-dialysis CKD patients is a true association and not an event secondary to the impaired urinary excretion of adiponectin.

Leptin, a 16 kDa peptide hormone and a risk factor for vascular calcification^5^, is also removed from plasma by the kidney. Similar to serum adiponectin, serum leptin is elevated in hemodialysis patients^6^.

We previously reported that hemodialysis patients exhibited a high prevalence of vascular calcification, and that the presence of AAC on plain radiographs was associated with either all-cause or cardiovascular mortality^7^.

In this cross-sectional study, to avoid the effect of reduced urinary excretion of adiponectin/leptin due to renal dysfunction on serum adiponectin/leptin levels, we examined the association between serum adiponectin/leptin and AAC in hemodialysis patients who do not have appreciable residual renal function.

## Results

### Clinical characteristics of patients, and comparisons between hemodialysis patients with and without AAC

Table 1 showed the characteristics of 101 hemodialysis patients, among whom 55 (54%) were positive for AAC on left abdominal X- ray radiographs. The serum level of adiponectin was 17.0 (11.3–24.9) µg/mL, which was approximately 3-fold higher than the reported value of 5.4 ± 2.3 µg/mL in male subjects without CKD, whereas the serum levels of leptin did not differ from those in subjects without CKD^8^. The serum adiponectin and leptin levels were significantly correlated in a negative and positive manner, respectively, with percentage body fat (adiponectin: ρ = −0.40, p < 0.001; leptin: ρ = 0.81, p < 0.001) in hemodialysis patients, which were consistent with the significant correlations reported previously in the general population^9, 10^.

**Table 1:** Clinical and biochemical characteristics of the study patients.

	All subjects	Without AAC	With AAC	p-value
Patients (n)	101	46	55	—
Age (years)	61 ± 11	58 ± 11	64 ± 10	0.01
Body weight (kg)	59.5 ± 9.5	60.4 ± 10.6	58.8 ± 8.5	0.40
Body mass index (kg/m^2^)	21.8 ± 3.0	21.8 ± 3.4	21.8 ± 2.6	0.99
Percentage body fat (%)	21.5 ± 7.0	21.4 ± 7.5	21.5 ± 6.7	0.99
Hemodialysis duration (years)	6.8 ± 2.9	6.6 ± 2.9	6.9 ± 2.9	0.65
Diabetes mellitus (n[%])	42 [42]	10 [22]	32 [58]	<0.001
Corrected calcium (mg/dL)	9.6 ± 0.7	9.6 ± 0.7	9.6 ± 0.8	0.57
Phosphate (mg/dL)	5.6 ± 1.1	5.6 ± 1.2	5.5 ± 1.1	0.54
Intact PTH (pg/mL)	108 (46–204)	139 (47–204)	100 (39–229)	0.78
hs-CRP (μg/dL)	113 (40–382)	73 (28–288)	122 (48–509)	0.07
Adiponectin (μg/mL)	17.0 (11.3–24.9)	15.4 (8.9–22.8)	18.8 (13.0–28.1)	0.03
Leptin (ng/mL)	4.3 (2.5–8.7)	4.2 (2.3–8.5)	4.7 (2.5–9.6)	0.77

Values are expressed as mean ± standard deviation, number, or median (Q_1_–Q_3_).

Conversion factors for units—calcium (mg/dL to mmol/L): × 2.2495, phosphate (mg/dL to mmol/L): ×0.3229.

AAC: abdominal aortic calcification, PTH: parathyroid hormone, hs-CRP: high-sensitivity C-reactive protein.

Various clinical parameters in hemodialysis patients were compared between those with and without AAC. AAC-positive hemodialysis patients were significantly older and had significantly higher prevalence of DM (p < 0.001) than their AAC-negative counterparts. Furthermore, AAC-positive patients showed a tendency towards having a higher serum high-sensitivity C-reactive protein (hs-CRP) (p = 0.07) than AAC-negative patients; however, the difference was not significant.

**Figure 1:**
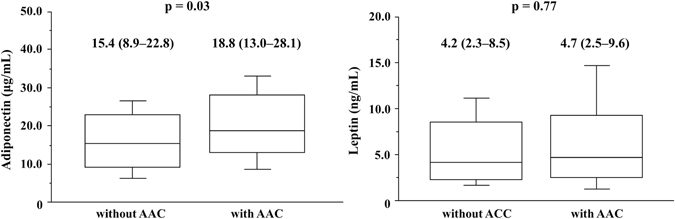
Relationship between abdominal aortic calcification (AAC) and serum adiponectin or leptin levels Serum adiponectin levels are significantly higher in patients with AAC than in those without (18.8 [13.0–28.1] µg/mL, n = 55 *vs*. 15.4 [8.9–22.8] µg/mL, n = 46; p = 0.03), whereas serum leptin levels are not significantly different between the two groups (4.7[2.5–9.6] ng/mL, n = 55 *vs*. 4.2 [2.3–8.5] ng/mL, n = 46; p = 0.77).

As shown in Fig. 1, AAC-positive patients exhibited significantly higher serum adiponectin than AAC-negative patients [18.8 (13.0–28.1) µg/mL, n = 55 *vs*. 15.4 (8.9–22.8) µg/mL, n = 46, p = 0.03], in contrast with the insignificant difference in serum leptin between the two groups of patients [4.7 (2.5–9.6) ng/mL, n = 55 *vs*. 4.2 (2.3–8.5) ng/mL, n = 46, p = 0.77].

### Multiple logistic regression analysis of the association between AAC and other factors

Multiple logistic regression analysis was employed to examine whether serum adiponectin was independently and significantly associated with the presence of AAC (Table 2). Model 1, which included log adiponectin, in addition to age, body weight, percentage body fat, hemodialysis duration, presence/absence of DM, serum calcium and phosphate levels, log intact parathyroid hormone (PTH), and log hs-CRP as independent variables, showed that log adiponectin, in addition to the presence/absence of DM, emerged as a significant and independent factor associated with the presence of AAC (odds ratio: 9.76, 95% confidence interval [CI]: 1.13–84.36, p = 0.04). Model 2, in which log adiponectin was replaced with log leptin, demonstrated that log leptin was not significantly associated with the presence of AAC (odds ratio: 3.13, 95% CI: 0.30–33.14, p = 0.34). Furthermore, even when log adiponectin and log leptin levels were simultaneously included as independent variables in model 3, the addition of log leptin did not affect the significant association of log adiponectin, as well as the presence/absence of DM, with the presence of AAC (odds ratio: 16.31, 95% CI: 1.70–156.41, p = 0.02).

**Table 2:** Multiple logistic regression analysis of factors associated with AAC.

	Model 1			Model 2			Model 3		
	OR	95% CI	p-value	OR	95% CI	p-value	OR	95% CI	p-value
Age (years)	1.04	0.99–1.10	0.13	1.04	0.99–1.10	0.18	1.04	0.99–1.10	0.12
Body weight (kg)	0.98	0.92–1.05	0.54	0.96	0.90–1.03	0.35	0.97	0.90–1.04	0.33
Percentage body fat (%)	1.03	0.95–1.13	0.50	0.96	0.85–1.09	0.80	0.95	0.84–1.10	0.47
Hemodialysis duration (years)	1.02	0.85–1.22	0.85	1.07	0.90–1.28	0.50	1.04	0.86–1.27	0.66
Diabetes (yes *vs*. no)	7.75	2.51–233.97	<0.001	7.52	2.53–22.36	<0.001	9.17	2.81–29.91	<0.001
Corrected calcium (mg/dL)	1.03	0.51–2.09	0.94	0.84	0.43–1.67	0.63	0.94	0.45–1.93	0.86
Phosphate (mg/dL)	1.00	0.65–1.55	0.98	1.07	0.70–1.63	0.76	1.04	0.67–1.61	0.86
Log{intact PTH (pg/mL)}	2.27	0.72–7.20	0.16	2.03	0.69–5.94	0.20	2.83	0.85–9.44	0.09
Log{hs-CRP (μg/dL)}	1.84	0.85–4.02	0.12	1.88	0.88–4.04	0.11	1.80	0.81–3.98	0.15
Log{leptin (ng/mL)}				3.13	0.30–33.14	0.34	8.54	0.61–119.00	0.11
Log{adiponectin (μg/mL)}	9.76	1.13–84.36	0.04				16.31	1.70–156.41	0.02
	R^2^ = 0.20, p < 0.001			R^2^ = 0.22, p < 0.001			R^2^ = 0.24, p < 0.001		

AAC: abdominal aortic calcification, OR: odds ratio, 95% CI: 95% confidence intervals, PTH: parathyroid hormone, hs-CRP: high-sensitivity C-reactive protein.

## Discussion

The present cross-sectional observational study demonstrated that higher serum adiponectin, but not leptin, was significantly and positively associated with the prevalence of AAC in male hemodialysis patients, suggesting higher serum adiponectin as a definite risk for AAC, independent of other risk factors for AAC, such as age, percentage body fat, hemodialysis duration, presence/absence of DM, and serum levels of calcium, phosphate, intact PTH, and hs-CRP. The presence of DM was significantly and positively associated with the prevalence of AAC in male hemodialysis patients, as we reported previously^11^.

Furthermore, the present study clearly demonstrated that the positive association of increased serum adiponectin with the presence of AAC thus far reported in pre-dialysis CKD patients could not be explained by the apparent increase of serum adiponectin due to the impaired renal excretion of adiponectin into urine, as hemodialysis patients without any appreciable residual renal function exhibited a significant, independent, and positive association between serum adiponectin and AAC. Previous studies showed that patients with CKD exhibited higher serum adiponectin due to impaired urinary adiponectin excretion, while they had a higher incidence of vascular calcification due to higher serum calcium × phosphate products, making the relationship between serum adiponectin and AAC significant and positive. Therefore, it remains difficult to distinguish whether increased serum adiponectin by itself might be a true risk factor that is intimately involved in the development of AAC, or simply a surrogate marker of renal dysfunction. However, the present study first determined that higher serum adiponectin could be independently and positively associated with the development of AAC in patients with CKD stage 5D without appreciable residual renal function. Because the enrolled hemodialysis patients had lost almost all residual renal function and thus could not produce a significant difference in the apparent increase of serum adiponectin due to impaired renal function, the present study clearly shows that increased serum adiponectin is by itself a positive factor associated with the prevalence of AAC and thus a definite risk for CVD in patients with CKD.

Epidemiologic studies, thus far performed mainly in patients with metabolic syndrome, have established the notion that serum adiponectin is negatively and independently associated with predictors for CVD risks and CVD events, by using various clinically useful markers for CVD risk, such as carotid intima-media thickness^12^, endothelial dysfunction of the coronary arteries, acute coronary syndrome, and multi-vessel coronary atherosclerosis^13^. Moreover, in a case-control study involving men without pre-existing CVD, higher adiponectin was found to be associated with a lower risk of myocardial infarction^2^. In basic studies, adiponectin-deficient mice developed arterial calcification *in vivo*, while adiponectin inhibited the differentiation of vascular smooth muscle cells into osteoblasts *in vitro* ^14^. However, contrary to the accepted hypothesis that reduced serum adiponectin is associated with an increased CVD risk or CVD mortality in men, such a relationship has been reported to be reversed, particularly in elderly subjects, in patients with pre-existing CVD^15^, and in pre-dialysis CKD patients^16^.

Among the various studies investigating the relationship between serum adiponectin and atherosclerosis in human subjects, only a few have investigated the relationship of serum adiponectin with vascular calcification. Serum adiponectin was not associated with calcified atherosclerotic plaques in patients with diabetes, with coronary artery calcification (in Caucasians)^17^, or with calcified aortic stenosis^18^. There are limited data suggesting that higher adiponectin levels are directly associated with vascular calcification in patients with CKD. The present study demonstrated that higher serum adiponectin levels were associated with a higher prevalence of AAC in hemodialysis patients. It is known that as renal dysfunction progresses to end-stage renal disease, the prevalence of vascular calcification becomes higher, and the presence and degree of AAC are independent predictors of future CVD events and mortality, as we previously reported^7^. Combining the data from our present study with the data from previous study, indicating the increase of serum adiponectin along with a decline in the estimated glomerular filtration rate in pre-dialysis CKD patients, it seems likely that the increase of serum adiponectin in pre-dialysis CKD patients might be involved in the development of AAC, leading to increased mortality. However, adiponectin has beneficial effects on cardiovascular cells through its antidiabetic, anti-inflammatory, and antiatherogenic action *in vitro* ^19^. The mechanism of why adiponectin positively induces AAC in hemodialysis patients might be explained by its effect on the nutritional and inflammatory status, which is a very common condition associated with the development of atherosclerosis including AAC. This condition, known as MIA (malnutrition, inflammation, atherosclerosis) -syndrome, is composed of malnutrition including decreases in body fat, which might be accompanied by an increase in serum adiponectin, and atherosclerosis including vascular calcification. Therefore, it is possible that the frequent occurrence of malnutrition in hemodialysis patients will reverse the association between serum adiponectin and AAC. However, in this study, the association between adiponectin and increased vascular calcification risk was not modified by body weight or percentage body fat. This suggests that decreases in body fat cannot fully account for the positive association between serum adiponectin and AAC. Alternatively, increased serum adiponectin might protect against the development of AAC in situations that stimulate AAC; however, its protective effect is insufficient^20^. It has been suggested that such counter-regulatory vascular protective mechanisms lead to increases in adiponectin levels. Finally, it was reported that adiponectin resistance may also induce these paradoxical responses to increased serum adiponectin in patients with AAC^21^.

Several studies have reported that increased serum leptin is associated with vascular calcification. For example, serum leptin was positively associated with higher coronary artery calcification in asymptomatic participants without DM^5^. Moreover, in Caucasians, serum leptin has been independently associated with coronary artery calcification after adjustment for age, sex, family history of CVD, DM status, exercise, and major cardiometabolic medications^17^. Furthermore, in basic research, leptin has been shown to promote osteoblast differentiation and mineralization in primary cultures of vascular smooth muscle cells^22^. However, in the present study, serum leptin was not associated with AAC.

This study has several limitations. First, because we determined the presence of AAC based on plain radiographs, which could not differentiate between intimal calcification (atherosclerosis) and medial calcification (arteriosclerosis), the presence of AAC in the present study did not equal to atherosclerosis. However, as not only intimal but also medial calcification is associated independently with mortality^23^, it provided a clinically relevant measure for CVD risk in hemodialysis patients, as we reported previously^7^. Second, all patients were Japanese men and the sample size was relatively small. Furthermore, because this was a cross-sectional observational study, we could not determine causality in the relationships between the studied variables. Lastly, recent studies have reported that there exist several adiponectin isoforms with different metabolic activities; we did not address this point in the present study.

In conclusion, high serum adiponectin is associated with AAC in hemodialysis patients. This association remains significant even after adjustment for age, percentage body fat, hemodialysis duration, DM, and serum levels of calcium, phosphate, intact PTH, hs-CRP, and leptin. The present study indicated that adiponectin is an independent factor that is positively associated with the prevalence of AAC in hemodialysis patients.

## Methods

### Subjects and study design

A cross-sectional observational study was conducted in 101 male Japanese hemodialysis patients in Shirasagi Hospital, Osaka, Japan, in 2003. Each patient provided written informed consent before being enrolled in the study. This study was approved by the Ethics Review Committee of Shirasagi Hospital^24^, and conducted in accordance with the principles of the Declaration of Helsinki. The enrolled patients were restricted to male to avoid the sex differences of serum adiponectin and/or leptin due to the sex differences in adiposity. Those who had acute illness, infection, or malignancy were excluded from the present study. The underlying kidney diseases of the enrolled patients were as follows: diabetic nephropathy (n = 42), chronic glomerulonephritis (n = 35), nephrosclerosis (n = 11), polycystic kidney disease (n = 2), other disease (n = 7), and unknown disease (n = 4). None of the patients had undergone parathyroidectomy or renal transplantation. All patients underwent three sessions of hemodialysis per week, each lasting for 4–5 hours, by using a hollow-fiber dialyzer, and a bicarbonate dialysate containing 3.0 mEq/L calcium. DM was diagnosed when the patient had a history of the disease or on the basis of the criteria of the American Diabetes Association^25^.

### Laboratory measurements

Blood samples were obtained just before the start of the dialysis session. Levels of serum albumin, calcium, and phosphate were measured by using routine laboratory methods. Aliquots of serum were stored at −20 °C while waiting for subsequent assay, and measurements were made immediately after thawing. The biochemical parameters of calcium metabolism were determined as previously described^26, 27^. Serum intact PTH levels were measured by using an electrochemiluminescence immunoassay kit (Elecsys PTH; Roche Diagnostics GmbH, Mannheim, Germany). Serum hs-CRP was measured through a nephelometric assay (SRL Inc., Tokyo Japan). Serum adiponectin levels were measured by using a human adiponectin ELISA kit (Otsuka Pharmaceuticals Co., Tokyo, Japan)^1^. Serum leptin levels were measured with a human leptin RIA kit (Millipore Co., Billerica, MA, USA)^28^.

### Body composition measurements

Total body fat was measured by means of dual X-ray absorptiometry (DXA; QDR-4500; Hologic, Waltham, MA, USA), and percentage body fat was calculated by using the built-in software of the DXA instrument, as previously reported^29–31^. The reproducibility of fat mass measurements with DXA was <2%, as previously reported^30^. Body mass index was defined as the patient’s weight in kilograms divided by the square of height in meters.

### Evaluation of AAC

We obtained plain radiographs of each patient’s left lateral abdomen in the standing position, by using KXO-50F (Toshiba Medical System, Tochigi, Japan) radiographic equipment at a voltage of 70 kV. One author (K.N.) evaluated whether there was any AAC between the levels of the first and fourth lumbar vertebrae. This author had been blinded to all other patient data, as previously reported^7^.

### Statistical analysis

Data are expressed as mean ± standard deviation, or median (25th-75th percentile). The normality of variables was assessed. Differences in mean and median values were evaluated by using Student’s t-test and the Mann–Whitney U test, respectively. We used the Spearman coefficient to assess the correlation between percentage body fat and serum levels of adiponectin or leptin. Multiple logistic regression analysis was performed to examine the influence of the following variables on AAC: age, body weight, percentage body fat, hemodialysis duration, DM, calcium levels, phosphate levels, intact PTH levels, hs-CRP levels, adiponectin levels, and leptin levels. Because the distributions of hs-CRP, intact PTH, adiponectin, and leptin were skewed, they were log-transformed to obtain a normal distribution. The odds ratios with 95% CIs, were calculated.

All statistical analyses were performed with StatView version 5.0 software (SAS Institute Inc., Cary, NC, USA); p-values < 0.05 were considered statistically significant.
